# Mesencephalic Astrocyte-Derived Neurotrophic Factor (MANF) Regulates Neurite Outgrowth Through the Activation of Akt/mTOR and Erk/mTOR Signaling Pathways

**DOI:** 10.3389/fnmol.2020.560020

**Published:** 2020-09-24

**Authors:** Wen Wen, Yongchao Wang, Hui Li, Hong Xu, Mei Xu, Jacqueline A. Frank, Murong Ma, Jia Luo

**Affiliations:** ^1^Department of Pathology, University of Iowa, Iowa City, IA, United States; ^2^Department of Pharmacology and Nutritional Sciences, University of Kentucky College of Medicine, Lexington, KY, United States; ^3^Department of Neurology, University of Kentucky College of Medicine, Lexington, KY, United States

**Keywords:** MANF, neurotrophic factor, neurite outgrowth, neuronal differentiation, Erk/mTOR, Akt/mTOR, protein synthesis

## Abstract

Neurite outgrowth is essential for brain development and the recovery of brain injury and neurodegenerative diseases. In this study, we examined the role of the neurotrophic factor MANF in regulating neurite outgrowth. We generated MANF knockout (KO) neuro2a (N2a) cell lines using clustered regularly interspaced short palindromic repeats (CRISPR)/Cas9 and demonstrated that MANF KO N2a cells failed to grow neurites in response to RA stimulation. Using MANF siRNA, this finding was confirmed in human SH-SY5Y neuronal cell line. Nevertheless, MANF overexpression by adenovirus transduction or addition of MANF into culture media facilitated the growth of longer neurites in RA-treated N2a cells. MANF deficiency resulted in inhibition of Akt, Erk, mTOR, and P70S6, and impaired protein synthesis. MANF overexpression on the other hand facilitated the growth of longer neurites by activating Akt, Erk, mTOR, and P70S6. Pharmacological blockade of Akt, Erk or mTOR eliminated the promoting effect of MANF on neurite outgrowth. These findings suggest that MANF positively regulated neurite outgrowth by activating Akt/mTOR and Erk/mTOR signaling pathways.

## Introduction

Neurite outgrowth is the first step for the formation of axons and dendrites, which are necessary components for the development of a functional neuronal network. Proper neurite outgrowth is not only important for the normal development of the nervous system, but may also facilitate the recovery of traumatic brain injury and neurodegenerative disorders such as Parkinson’s disease (PD), Alzheimer’s disease (AD), and Amyotrophic lateral sclerosis (ALS) (Gouel et al., 2019; Houlton et al., 2019). Neurite outgrowth requires the synthesis and reorganization of cytoskeleton proteins including actin and microtubules (Rodriguez et al., 2003; Flynn, 2013). The dynamic of cytoskeleton elongation, branching, and retraction is regulated by various factors such as transcription factors that governs gene expression for neuron differentiation (Paolino et al., 2018) and cell adhesive molecules that interact with and remodel cytoskeleton and activate gene expression (Missaire and Hindges, 2015; Leshchyns’ka and Sytnyk, 2016). Moreover, neurite outgrowth is also regulated by neurotrophic factors (NTF) that activate cell signaling pathways for neuron differentiation and migration, and support neuron survival and synaptic function (da Silva and Dotti, 2002; Cui, 2006).

Mesencephalic astrocyte-derived neurotrophic factor (MANF), also known as arginine-rich, mutated in early stage tumors (Armet), together with cerebral dopamine neurotrophic factor (CDNF) form a novel family of neurotrophic factors in vertebrate (Petrova et al., 2003; Lindholm et al., 2007). MANF is an endoplasmic reticulum (ER)-stress inducible protein, and its expression and secretion can be regulated by ER-stress (Mizobuchi et al., 2007; Oh-Hashi et al., 2012). MANF is broadly expressed in multiple developing and mature tissues including the central nervous system. In rodent brain, MANF is widely expressed in all brain regions during early developmental stages and declined gradually when the brain matures except for certain brain regions in the cerebral cortex, hippocampus, and cerebellar Purkinje cells, suggesting the role of MANF in neuron differentiation (Lindholm et al., 2008; Wang et al., 2014; Yang et al., 2014; Danilova et al., 2019). Increasing evidence have shown that MANF promotes the development and survival of neurons in both normal and pathological conditions (Petrova et al., 2003; Palgi et al., 2009; Voutilainen et al., 2009; Airavaara et al., 2010; Chen et al., 2012). MANF is required for dopaminergic neuron survival in *Drosophila* and zebrafish (Palgi et al., 2009; Chen et al., 2012), and selectively protects dopaminergic neuron in rat medial ventral mesencephalon cell cultures *in vitro* (Petrova et al., 2003). In rat PD model induced by 6-hydroxydopamine (6-OHDA), intrastriatally injection of MANF protects nigrostriatal dopaminergic nerves from degeneration (Voutilainen et al., 2009). Overexpression of MANF ameliorates the loss of Purkinje cells in a mouse model of spinocerebellar ataxia (Yang et al., 2014) and promotes neural progenitor cells migration and differentiation in a rat cortical stroke model (Tseng et al., 2017a). In addition, MANF is also reported to facilitate retinal ganglion cells and photoreceptor cells regeneration in the retina by regulating neuroinflammation and immune response (Neves et al., 2016; Gao et al., 2017; Lu et al., 2018). Similarly, CDNF has also been reported to be neuroprotective in animal models of PD, AD, and periphery nerve injury (Lindholm et al., 2007; Cheng et al., 2013; Kemppainen et al., 2015).

Recently, a study using conventional MANF knockout mice (Manf ^–/–^) revealed that MANF may be involved in neurite outgrowth (Tseng et al., 2017b). Manf ^–/–^ cortex showed decreased dendrite and axon length, while MANF deficient neural stem cells (NSCs) have impaired ability to grow neurites in culture. However, the mechanism and cellular signaling involved in MANF regulation neurite outgrowth remain unclear. In the present study, we used mouse neuro2a (N2a) cells to investigate the mechanisms underlying MANF regulation of neurite outgrowth. N2a cells are a neuronal cell line model widely used for studying neuronal differentiation; they differentiate into neuron-like cells in response to stimulants such as serum starvation, retinoic acid (RA), and cyclic adenosine monophosphate (cAMP) treatments (Salto et al., 2015). We demonstrated that MANF was required for RA-induced neurite outgrowth. MANF regulation of neurite outgrowth was mediated by Akt/mTOR and Erk/mTOR signaling pathways and protein synthesis.

## Materials and Methods

### Materials

The following cells and materials were used: N2a (CCL-131), SH-SY5Y (CRL-2266) and HEK293 (CRL-1573) cells were from ATCC (Manassas, VA, United States); MEM (11095-080), high glucose DMEM (10569-010), L-methionine free DMEM (21013-024), FBS, antibiotic-antimycotic (15240112), GeneArt Genomic Cleavage Detection Kit (A24372), and Lipofectamine 3000 Reagent (L3000008) were from Life Technologies (Carlsbad, CA, United States); all-*trans* RA (R2625), crystal violet acetate (C5042), MTT (M5655), anhydrous DMSO (276855), DAPI (D9542), Akt activator SC79 (SML0749), and mTOR activator MHY1485 (SML0810) were from Sigma-Aldrich (St. Louis, MO, United States); PFA (15714) was from Electron Microscopy Sciences (Hatfield, PA, United States); recombinant human MANF (hMANF) (MANF-536H) was from Creative BioMart (New York, NY, United States); control CRISPR/Cas9 plasmid (sc-418922), mouse ARP double nickase plasmid (sc-428989-NIC), UltraCruz transfection reagent (sc-395739), plasmid transfection medium (sc-108062), and Akt inhibitor MK-2206 dihydrochloride (sc-364537) were from Santa Cruz Biotechnology (Dallas, TX, United States); Erk activator PDBu (12808), Erk inhibitor PD98059 (9900), and mTOR inhibitor Torin 1 (14379) were from Cell Signaling Technology (Beverly, MA, United States); pGEM-T-easy vector (A1360) was from Promega (Madison, WI, United States); 10-beta competent *E. coli* (C3019I) was from New England Biolabs (Ipswich, MA, United States); scrambled siRNA-GFP lentivector (LV015-G), Manf siRNA-GFP lentivector (279970940495), MANF-HA adenovirus (mouse) (279970540200), and CMV Null control adenovirus (000047A) were from Applied Biological Materials (Richmond, BC, Canada); PureLink Expi Endotoxin-free Maxi Plasmid Purification Kit (A31231) was from Thermo Fisher Scientific (Waltham, MA, United States); VECTASHIELD mounting medium (H-1400 and H-1500) was from Vector Laboratories (Burlingame, CA, United States); *DC* protein assay kit (5000112) was from Bio-Rad Laboratories (Hercules, CA, United States); Click-iT HPG Alexa Fluor Protein Synthesis Assay Kits (C10428) was from Invitrogen (Grand Island, NY, United States).

The following antibodies were used: anti-α-tubulin (T5168, Sigma-Aldrich); anti-ARMET/ARP (MANF) (ab67271 for C-terminus and ab67203 for N-terminus, Abcam, Cambridge, MA, United States); anti-HA-Tag (CST 3724), anti-phospho-Akt (Ser473) (CST9271), anti-Akt (CST9272), anti-phospho-Erk1/2 (CST 9101), anti-Erk1/2 antibody (CST 9102), anti-phospho-mTOR (Ser2448) (CST2971), anti-mTOR (CST2972), anti-phospho-p70 S6 (CST9204), anti-p70 S6 (CST2708), anti-Cas9 (CST 14697), and anti-β-Actin (CST3700) antibodies were all from Cell Signaling Technology; secondary antibodies conjugated to horseradish peroxidase (NA931V and NA934V) were from GE Healthcare Life Sciences (Pittsburgh, PA, United States); Alexa-488 conjugated anti-mouse (A21202), Alexa-594 conjugated anti-mouse (A11005) and Alexa-594 conjugated anti-rabbit antibodies (A11012) were from Life Technologies.

### Cell Culture and Differentiation

Neuro2a cells and HEK293 cells were cultured in MEM supplemented with 10% FBS and 1% antibiotic-antimycotic at 37°C in 5% CO_2_ in a humidified incubator (Symphony 5.3A, Thermo Scientific). SH-SY5Y cells were cultured in DMEM supplemented with 10% FBS and 1% antibiotic-antimycotic. To induce neurite outgrowth, N2a or SH-SY5Y cells were plated in 6-well plates at a density of 5 × 10^4^ cells per well for 24 h. Then growth medium was carefully removed and replaced with differentiation media of an equal volume of MEM or DMEM supplemented with 2% FBS at the present of 10 μM all-*trans* RA for 3 days unless otherwise stated. Recombinant human MANF (hMANF) was added into differentiation media at a final concentration of 100 ng/ml. Differentiation media was changed freshly every other day to ensure RA effectiveness. Neurite was defined as the extension of neuronal processes greater than two cell body diameters in length. To quantify neurite length, at least five random fields were imaged for each well using Olympus BX51 light microscope with the 40X objective. The five random fields were selected in an X manner with four fields at the corners and one field in the center of the well. Cells were either imaged alive or fixed in 4% PFA for 10 min then stained with 0.5% crystal violet for 15 min at room temperature. Neurite length was measured from the center of the cell body to the tip of the longest neurite using the CellSens imaging software (Olympus, Tokyo, Japan). Morphometrics analysis was performed independently by two or more different investigators. To quantify the percentage of cells with neurite, the total cell numbers and cells with neurite in each field were counted with ImageJ.

### CRISPR Transfection and MANF Knockout Single Cell Colony Isolation

**FIGURE 1:**
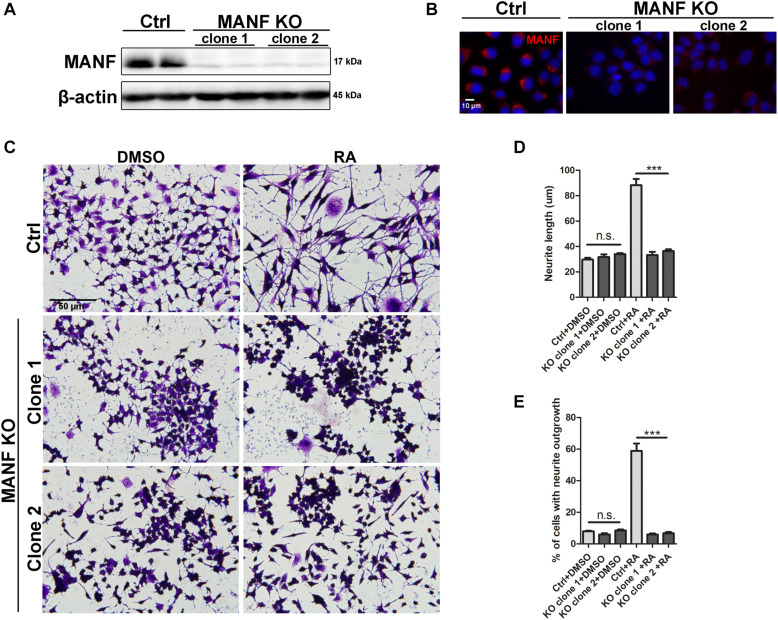
Loss of MANF inhibits RA-induced neurite outgrowth. Stable N2a cell lines of MANF knockout (KO) were established as described in the Materials and Methods. **(A)** Protein was collected from control and MANF KO cell lysates and analyzed by immunoblotting with MANF antibody. β-actin was used as a loading control. The size of the proteins (kDa) was labeled next to each band. **(B)** Immunofluorescent staining showed MANF expression in control cells and no signal in MANF KO cells. Cell nuclei were stained by DAPI. **(C)** Representative images of control and MANF KO cells treated with DMSO or RA for 3 days. Cells were fixed and stained with crystal violet for visualization. Average neurite length **(D)** and percentage of cells that bear neurites **(E)** were measured, and analyzed by One-way ANOVA. In cases where significant differences were detected, specific *post hoc* comparisons between treatment groups were examined with the Tukey’s test. ***P < 0.0001, n.s. not statistically significant. The data were expressed as the mean ± SEM of three independent experiments.

Control CRISPR/Cas9 plasmid was referred to control CRISPR thereafter and mouse ARP double nickase plasmid was referred to MANF CRISPR thereafter. Control CRISPR has a GFP marker for visually confirmation of successful transfection. MANF CRISPR consists a pair of plasmids both targeting the exon 2 of mouse Manf gene. One plasmid contains a puromycin resistance gene, the other one has a GFP marker. N2a cells were seeded onto 6-well plate to grow to 70% confluence and were transfected with 1 μg of either control or MANF CRISPR using UltraCruz transfection reagent and plasmid transfection medium according to the manufacture’s protocol. GFP-positive cells were selected by flow cytometry 48 h after the transfection. MANF CRISPR transfected cells that were GFP-positive were further selected by 1 μg/ml puromycin for 5 days, then diluted and plated onto 10 cm culture dishes at 1 × 10^3^ cells/dish to form single cell colonies. Once single cell colonies were formed and large enough, they were transferred using sterile 10 μl pipette tips to 24-well plates and then 6-well plates to expand. The presence of genomic insertions or deletions (indels) in each single cell colony were detected using the GeneArt Genomic Cleavage Detection Kit (Life Technologies) according to the manufacture’s protocol. Among fourteen single cell colonies we tested, five appeared to have genomic indels. To confirm biallelic MANF knockout, we tested at genomic DNA and protein level. Genomic DNA was isolated from these five colonies. MANF was cloned by PCR using primers 5′-AGTTTTTTCCAGGGGAAATGG-3′ and 5′-ACCCACTACTTTCTCTCTCAG-3′, and then cloned into the pGEM-T-easy vector (Promega) and transformed into 10-beta competent *E. coli* (New England Biolabs) for sequencing. Protein was also extracted from each colony and subjected to immunoblot to test the presence of MANF protein. Ultimately, we confirmed 2 colonies that have biallelic MANF KO. To rule out the possibility of clonal selection, both colonies were tested in morphometric analysis in Figure 1 and protein expression analysis in Figures 6, 7. Since similar results were revealed between the two clones, we used clone 1 for all other analysis.

### siRNA Transfection

Scrambled siRNA-GFP and Manf siRNA-GFP lentivector plasmids were purchased from Applied Biological Materials (Richmond, BC, Canada) with the MANF siRNA target sequence 5′-TCAAAGACAGAGATGTCACATTTTCACCA-3′ cloned into the piLenti-siRNA-GFP plasmid backbone. The siRNA is driven by the U6 promotor followed by the GFP driven by the CMV promotor. The plasmids were subcloned and amplified in 10-beta competent *E. coli* cells (New England Biolabs), and purified using PureLink Expi Endotoxin-free Maxi Plasmid Purification Kit (Thermo Fisher Scientific). Either 2.5 μg of scrambled siRNA or Manf siRNA lentivector plasmids were transfected into N2a or SH-SY5Y cells using Lipofectamine 3000 Reagent (Life Technologies) according to the manufacturer’s instruction. Cells were subject to subsequent analysis.

### Adenovirus Transduction

Crude viral stock of MANF-HA adenovirus (mouse), referred to AD-MANF thereafter, and CMV Null control adenovirus, referred to AD-vector thereafter (Applied Biological Materials) were prepared at titer ranging from 1 × 10^7^ to 1 × 10^8^ pfu/ml by manufacture and then amplified in HEK293 cells according to the manufacture’s instruction. Briefly, 3 × 10^6^ HEK293 cells were plated in 10 cm dishes and incubated with 100 μl crude viral stock until 90% of the cells were rounded up and detached. Adenovirus-containing HEK293 cells were collected with culture media and placed at −80°C for 30 min and then in 37°C water bath for 15 min to thaw. Repeat the freeze and thaw for 3 times. Cell debris were pelleted at 3000 rpm for 15 min and supernatant with viral particles were used to transduce N2a cells. N2a cells were transduced with either CMV Null control or MANF-HA adenovirus by incubating 120 μl per well in 6-well plate for 3 h. Three hours later, culture media with adenovirus were removed and replaced with fresh growth media. Cells were subject to subsequent analysis.

### Immunocytochemistry

Neuro2a or SH-SY5Y cells were seeded onto 24-well plates with sterile coverslip on the bottom coated with 10 μg/ml fibronectin. After differentiation, cells were fixed with 4% PFA and permeabilized in 0.25% Triton X-100 in PBS for 10 min at room temperature. Then cells were blocked with 1% BSA/2% goat serum/PBS for 30 min and incubated with anti-α-tubulin antibody (1:1000) 1 h at room temperature. After washing with PBS, cells were incubated with Alexa fluor-conjugated secondary antibodies at a dilution of 1:200. Cells on the coverslip were counterstained with DAPI and sealed with VECTASHIELD mounting medium (Vector Laboratories). Fluorescence images were obtained using the Olympus IX81 inverted fluorescent microscope (Olympus).

### Protein Extraction and Immunoblotting

Protein was extracted from N2a or SH-SY5Y cells as previously described (Xu W. et al., 2018). Briefly, cells were washed with ice cold PBS and lysed on ice for 15 min in RIPA buffer containing 150 mM NaCl, 1 mM ethylene glycol-bis(beta-aminoethyl ether)-N,N,N′,N′-tetraacetic acid (EGTA), 50 mM Tris–HCl (pH 7.5), and 0.5% Nonidet P-40 (NP-40), 0.25% sodium dodecyl sulfate (SDS), with freshly added protease inhibitors of 5 μg/ml leupeptin, 5 μg/ml aprotinin, 3 mM sodium orthovanadate, and 0.3 mg/ml phenylmethanesulfonyl fluoride (PMSF). Lysed cells were centrifuged at 10,000 × *g* for 30 min at 4°C, and the supernatant fraction was collected. Protein concentration was determined using the *DC* protein assay (Bio-Rad Laboratories) according the manufacture’s instruction.

Immunoblotting was performed as previously described (Li et al., 2019; Pillai-Kastoori et al., 2020a, b). In brief, 20–30 μg protein samples were separated by SDS-PAGE on 12% polyacrylamide gels by electrophoresis. Separated proteins were then transferred to nitrocellulose membranes and blocked in 5% BSA/1xTBS/0.05% Tween-20 for 1 h at room temperature prior to incubation with primary antibodies at 4°C overnight. The primary antibodies used and the final dilutions were as follows: anti-ARMET/ARP (MANF), anti-HA-Tag, anti-phospho-Akt (Ser473), anti-Akt, anti-phospho-Erk1/2, anti-Erk1/2 antibody, anti-phospho-mTOR (Ser2448), anti-mTOR, anti-phospho-p70S6, anti-p70S6, anti-Cas9, and anti-β-actin (1:1000). Subsequently, membranes were washed with TBST and incubated with secondary antibodies conjugated to horseradish peroxidase (1:5000). Blots were developed using the Amersham ECL Prime Western Blotting Detection Reagent (GE Healthcare Life Sciences). The density of immunoblotting was quantified using Image Lab software (Bio-Rad Laboratories).

### MTT Assay

To examine cell metabolic activity, 3-(4,5-dimethylthiazol-2-yl)-2,5-diphenyltetrazolium bromide (MTT) assay was used. Cells were seeded on 96-well plates at 2 × 10^3^ cells per well. At times indicated, MTT was added to each well at the final concentration of 500 μg/ml and incubated at 37°C for 2 h. After the incubation, media were carefully removed and 100 μl DMSO was added to each well to dissolve the MTT formazan. Plates were read using the Beckman Coulter DTX 880 Multimode Detector plate reader (Analytical Instruments, Golden Valley, MN, United States) at the wavelength of 595 nm.

### Pharmacological Inhibition or Activation of Akt, Erk, and mTOR

Cells were preincubated with Akt activator SC79 (2.5 μM), Erk activator PDBu (2 μM), mTOR activator MHY1485 (5 μM) and Akt inhibitor MK-2206 dihydrochloride (2.5 μM), Erk inhibitor PD98059 (50 μM), mTOR inhibitor Torin 1 (50 nM) for 30 min and then treated with RA as described above.

### Click-iT Homopropargylglycine (HPG) Protein Synthesis Assay

To detect the rate of protein synthesis, we utilized the Click-iT HPG Alexa Fluor Protein Synthesis Assay Kits (Invitrogen). Control and MANF KO N2a cells were seeded onto either fibronectin coated 96-well plates or fibronectin coated 24-well plates with sterile coverslip on the bottom. Protein synthesis was detected for cells treated with RA or DMSO control at 24, 48, and 72 h. An hour before the click-it reaction, media was replaced with DMEM without L-methionine (Life Technologies) to deplete methionine. Then cells were incubated with 50 μM HPG in L-methionine free DMEM media for 1 h and fixed with 4% PFA in PBS. After permeabilization in 0.5% TritonX-100 in PBS, cells were incubated in Click-iT reaction cocktail at room temperature for 30 min in dark and then cell nuclei were counter stained with DAPI. The rate of HPG incorporation in 96-well plates was examined using the Beckman Coulter DTX 880 Multimode Detector plate reader (Analytical Instruments) at the wavelength of 535 nm for the fluorescent intensity. Coverslips were removed from 24-well plates and mounted onto slides and images were taken using the Olympus IX81 inverted fluorescent microscope (Olympus). Images were analyzed using ImageJ for calculating the percentage of cells without HPG incorporation.

### Statistical Analysis

All analyses were performed using the GraphPad Prism version 7 software. Data were expressed as mean ± SEM of three independent experiments in each group. Differences among experimental groups were analyzed by Student *t*-test, one-way ANOVA or two-way ANOVA with *p* < 0.05 being considered statistically significant. In cases where significant differences were detected, specific comparisons between treatment groups were examined with the Tukey’s *post hoc* test for one-way ANOVA or Bonferroni’s *post hoc* test for two-way ANOVA.

## Results

### MANF Deficiency Inhibits RA-Induced Neurite Outgrowth

Mesencephalic astrocyte-derived neurotrophic factor knockout (KO) N2a cells were generated by transfection of MANF CRISPR/Cas9. Two single cell colonies with biallelic MANF knockout were isolated. Immunoblotting and immunocytochemistry revealed that MANF protein was not present in both clones of MANF KO N2a cells (Figures 1A,B). To examine if MANF is required for neurite outgrowth in N2a cells, control and KO cells were treated with 10 μM RA or DMSO control in media with 2% FBS for 3 days. The length of neurite and percentage of cells with neurite was analyzed. Strikingly, among 500–800 cells analyzed in three independent experiments, significantly fewer numbers of MANF KO cells have grown neurite and the length of neurite was significantly shorter compared to control cells. The treatment of RA results in about 60% control cells to grow neurites, but only ∼6% KO cells bear neurites (Figures 1C,E). The average neurite length after 3 days of RA treatment in KO cells was only 33.27 ± 9.95 μm for clone 1 and 36.42 ± 12.41 μm for clone 2, compared to 88.28 ± 20.59 μm for control cells (Figures 1C,D).

**FIGURE 2:**
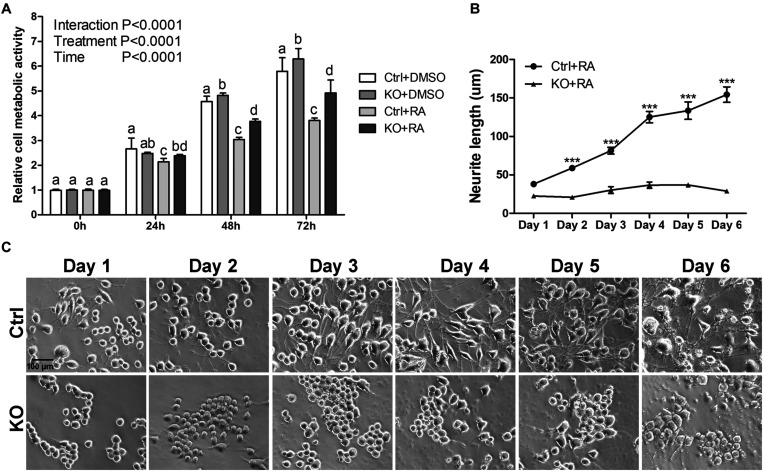
The reduction of neurite outgrowth in MANF KO cells is neither due to toxicity nor delayed process. **(A)** Cell metabolic activity at 0, 24, 48, and 72 h of RA treatment was tested by MTT assay. Different letters above columns indicate significant differences between treatments as tested by two-way ANOVA followed with the Bonferroni’s *post hoc* test (*P* < 0.05). The data were expressed as the mean ± SEM of three independent experiments. **(B)** Average neurite length of control and MANF KO cells after 6 days of RA treatment was determined and analyzed by Two-way ANOVA followed with the Bonferroni’s *post hoc* test, ***P < 0.001. The data were expressed as the mean ± SEM of three independent experiments. **(C)** Representative images of control and MANF KO cells treated with RA.

To determine whether the reduction of neurite outgrowth in MANF KO cells was a consequence of general toxicity due to MANF knockout or RA treatment, we measured the cellular metabolic activity of clone 1 by MTT assay. We observed a steady increase of viable and metabolically active cells for both DMSO treated control and KO cells, indicating that MANF knockout does not affect N2a cell metabolic activity (Figure 2A). Interestingly, KO cells showed higher cell metabolic activity at 48 and 72 h compared to control cells. In addition, we noticed that RA treatment slowed down cell growth and RA-treated control cells have significantly slower proliferation rate compared to other groups, which was in line with the fact that the majority of RA treated control cells ceased proliferation and underwent differentiation (Figure 2A).

To test the possibility that MANF KO cells may have delayed neurite outgrowth in response to RA treatment, we prolonged cells in differentiation media for 6 days and measured neurite length each day. For control cells, we observed a gradual increase in neurite length and by 6 days the neurite length reached to 154.45 ± 43.47 μm. However, for KO cells (clone 1 was used for this analysis), we only observed a subtle neurite growth to 36.94 ± 14.11 μm and it started to decline after 5 days (Figures 2B,C).

**FIGURE 3:**
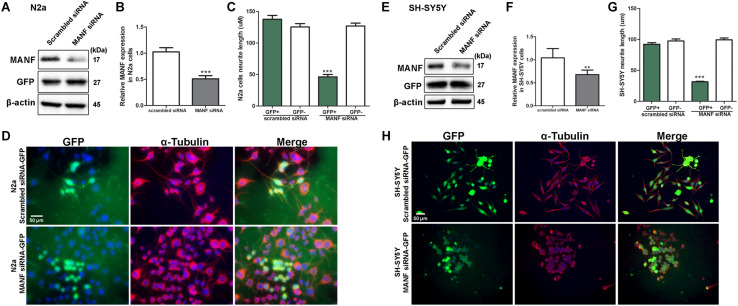
MANF knockdown by siRNA inhibits RA-induced neurite outgrowth. N2a and SH-SY5Y cells were transfected with MANF siRNA-GFP or scramble siRNA-GFP construct as described in the section “Materials and Methods.” **(A,E)** 24 h after the transfection, cells were lysed and analyzed by immunoblotting with MANF and GFP antibodies. β-actin was used as a loading control. The size of the proteins (kDa) was labeled next to each band. **(B,F)** MANF protein levels were quantified and normalized to β-actin. The data were analyzed by Student’s *t* test, ***P < 0.0001. The data were expressed as the mean ± SEM of three independent experiments. **(C,G)** Average neurite length in scramble siRNA and MANF siRNA transfected GFP+ cells after 3 days of RA treatment was determined and analyzed by Student’s *t* test, ***P < 0.0001. The data were expressed as the mean ± SEM of three independent experiments. **(D,H)** Representative fluorescent images revealed GFP expression in cells with successful siRNA transfection (Green). Neurites were visualized by immunofluorescent labeling of α-tubulin (Red). Cell nuclei were stained by DAPI. **P < 0.01.

To confirm that the inhibition of neurite outgrowth was specific to MANF deficiency, we performed an alternative approach of using siRNA to knockdown MANF in N2a and SH-SY5Y cells and compared neurite length between MANF siRNA and scramble siRNA transfected cells. MANF siRNA sufficiently reduced about 50% MANF expression in N2a cells (Figures 3A,B) and 30% in SH-SY5Y cells (Figures 3E,F). Since both scrambled siRNA and MANF siRNA plasmids contain GFP, we were able to use the expression of GFP as an indicator for successful transfection and analyze neurite length in GFP positive cells. Consistent with MANF CRISPR KO cells, siRNA MANF knockdown resulted in reduced neurite outgrowth in response to RA treatment compared to control cells as shown by α–tubulin labeling (Figures 3C,D,G,H). Scramble siRNA transfected N2a cells send out neurite at an average of 137.69 ± 25.06 μm in respond to RA treatment, while MANF siRNA transfected N2a cells only grow neurite at 46.25 ± 20.87 μm. Similarly, scramble siRNA transfected SH-SY5Y cells send out neurite at an average of 92.19 ± 27.55 μm in respond to RA treatment, while MANF siRNA transfected SH-SY5Y cells only grow neurite at 31.68 ± 7.71 μm. All these data above suggested that MANF deficiency inhibited neurite outgrowth.

### MANF Overexpression Facilitates RA-Induced Neurite Outgrowth

**FIGURE 4:**
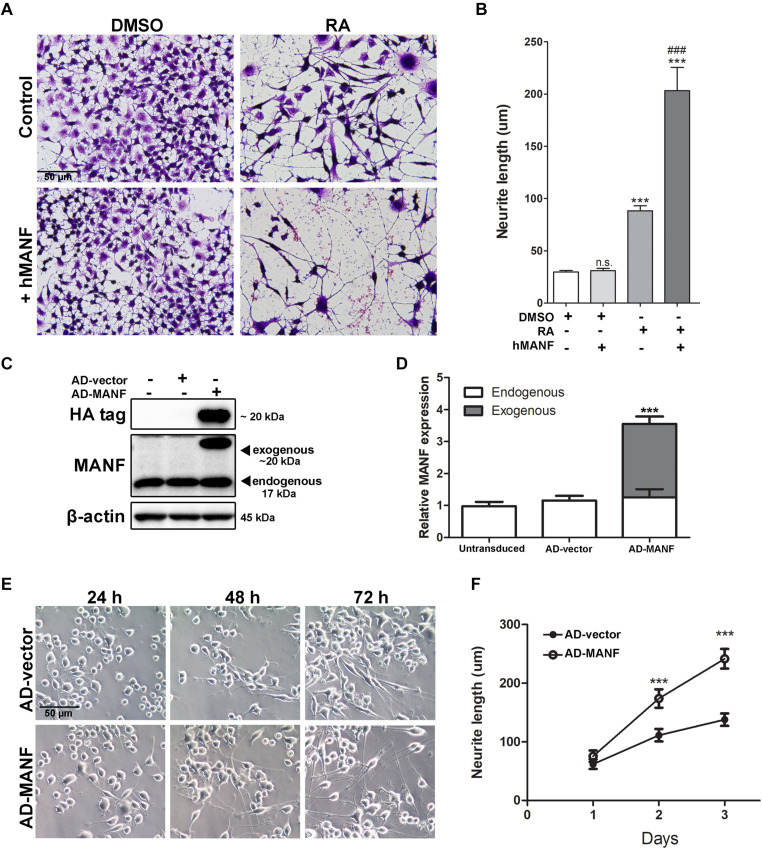
MANF overexpression facilitates RA-induced neurite outgrowth. **(A)** Representative images of N2a cells treated with DMSO or RA for 3 days with or without addition of recombinant hMANF (100 ng/ml). Cells were fixed and stained with crystal violet for visualization. **(B)** Average neurite length was measured, and analyzed by One-way ANOVA followed with the Tukey’s *post hoc* test, ***P < 0.0001 and n.s. not statistically significant compared to DMSO treated cells, ^###^*P* < 0.0001 compared to RA treated cells. The data were expressed as the mean ± SEM of three independent experiments. **(C)** Cells were lysed 48 h after adenovirus transduction and subjected to immunoblot to determine levels of MANF and HA tag expression. The size of the proteins (kDa) was labeled next to each band. **(D)** Endogenous and exogenous MANF protein levels were quantified and normalized to β-actin. The data were analyzed by Student’s *t* test, ***P < 0.0001. The data were expressed as the mean ± SEM of three independent experiments. **(E)** Representative images of AD-vector and AD-MANF transduced cells treated with RA for 24, 48, and 72 h. **(F)** Average neurite length of AD-vector and AD-MANF transduced cells after RA treatment was determined, and analyzed by Two-way ANOVA followed with the Bonferroni’s *post hoc* test, ***P < 0.001. The data were expressed as the mean ± SEM of three independent experiments.

Mesencephalic astrocyte-derived neurotrophic factor is mostly located in the lumen of endoplasmic reticulum (ER), but it can also be secreted under pathological conditions such as ER-stress (Apostolou et al., 2008; Glembotski et al., 2012). Since it is still unclear whether the biological function of MANF is through its intracellular form or secreted form, we performed two experiments to test whether MANF can facilitate neurite outgrowth in response to RA treatment through its intracellular or extracellular form, respectively. First, we examined whether addition of extracellular MANF in the culture media can induce neurite outgrowth in N2a cells. We found that the addition of MANF (100 ng/ml) into the culture media alone did not induce N2a cells to grow neurites (Figures 4A,B). However, when MANF was combined with RA, cells grow long neurites that reached 203.26 ± 71.02 μm after 3 days, which was significantly longer than cells treated with RA alone (88.28 ± 20.59 μm) (Figures 4A,B). This result suggested that extracellular MANF can facilitate neurite outgrowth in response to RA.

Then, we induced the overexpression of MANF intracellularly by adenovirus (AD) transduction. Adenovirus transduction did not affect endogenous MANF expression when compared to untransduced cells (Figures 4C,D). Immunoblotting detected that AD-MANF transduced cells show robust exogenous MANF expression which migrate slower than the endogenous MANF due to the presence of HA-tag (Figures 4C,D). The expression of exogenous MANF protein in AD-MANF transduced N2a cells peaked around 48 h after transduction and started to decline afterward (Supplementary Figure S1). Twelve hours after the transduction, cells were incubated in differentiation media for 3 days and neurite length was measured each day. We found that AD-MANF transduced N2a cells grow significantly longer neurites (173.83 ± 52.32 μm) compared to AD-vector transduced control cells (111.20 ± 33.83 μm) starting at 2 days. By day 3, AD-MANF transduced cells still have significantly longer neurites (241.66 ± 58.16 μm) than control cells (137.82 ± 37.29 μm) (Figures 4E,F). As a result, we concluded that both extracellular and intracellular MANF can facilitate N2a cell neurite outgrowth in response to RA stimulation.

### Addition of MANF in Culture Media Fails to Rescue Neurite Outgrowth Defects in MANF KO Cells in Response to RA Treatment

Since both intracellular and extracellular MANF facilitates RA-induced neurite outgrowth in control N2a cells, we want to test whether addition of MANF can rescue the neurite outgrowth defects in MANF KO cells. We first tried to overexpress MANF intracellularly in MANF KO cells. Unfortunately, we were unable to reintroduce MANF back into MANF KO cells by AD-MANF transduction for two possible reasons. First, the transduction efficiency was much lower in KO cells compared to control cells. As shown in Supplementary Figure S2, we were able to detect HA signal in 85% of control cells with correspondence strong exogenous MANF expression. However, only 31% of KO cells showed weak HA expression and no exogenous MANF was detected. The other reason why we were unable to reintroduce MANF expression in KO cells was that we still detected high level of Cas9 protein expression in KO cells (Supplementary Figure S2D), indicating the integration of the MANF CRISPR/Cas9 construct into the genome of the cells. Since the MANF CRISPR target sites were located within exon 2 of mouse *Manf* gene, the presence of MANF CRISPR/Cas9 in the cells was able to interfere with the expression *Manf* gene carried by AD-MANF, further inhibit the expression of exogenous MANF.

**FIGURE 5:**
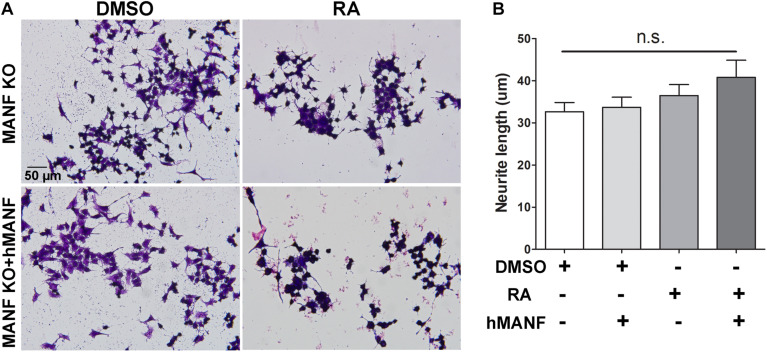
Addition of extracellular hMANF fails to rescue the neurite outgrowth defects in MANF KO cells. **(A)** Representative images of MANF KO N2a cells treated with DMSO or RA for 3 days with or without addition of recombinant hMANF (100 ng/ml). Cells were fixed and stained with crystal violet for visualization. **(B)** Average neurite length was measured, and analyzed by One-way ANOVA followed with the Tukey’s *post hoc* test, n.s. not statistically significant. The data were expressed as the mean ± SEM of three independent experiments.

As a result, we were only able to test whether addition of extracellular MANF can rescue the neurite outgrowth defect in MANF KO cells. MANF KO cells were treated with DMSO or RA with or without addition of recombinant hMANF (100 ng/ml) in the culture media for 3 days (Figure 5). As before, neither RA nor hMANF can induce MANF KO cells to grow neurites. In addition, the combination of RA+hMANF also failed to induce neurite outgrowth in MANF KO cells, suggesting that addition of extracellular MANF is not sufficient to rescue the neurite outgrowth defects in MANF KO cells in response to RA.

### MANF Regulates RA-Induced Neurite Outgrowth Through the Activation of Akt/mTOR and Erk/mTOR Signaling Cascades

**FIGURE 6:**
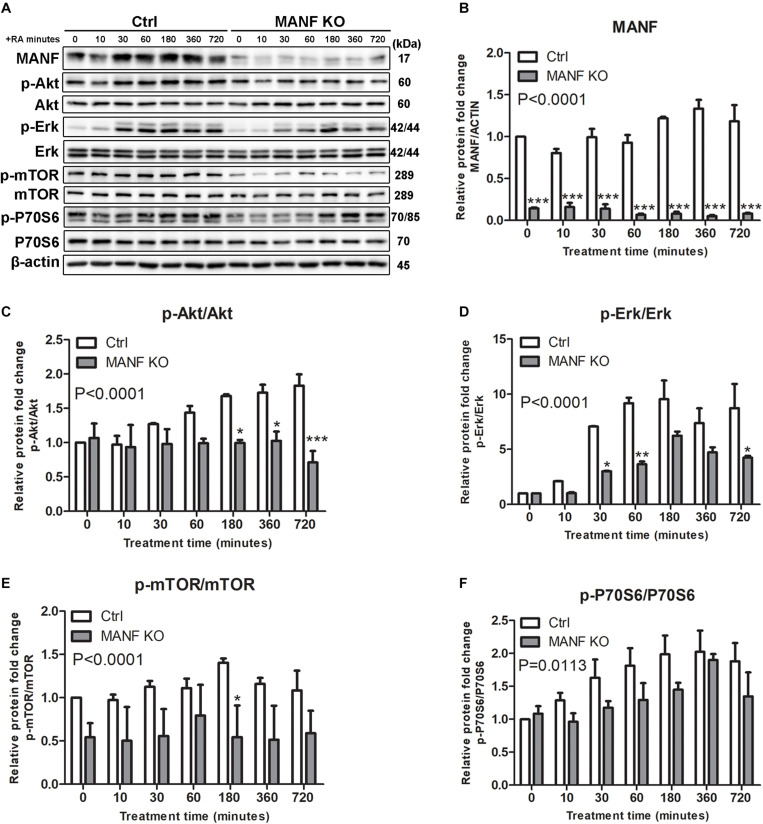
MANF knockout reduces RA-induced activation of Akt/mTOR and Erk/mTOR signaling pathways. **(A)** Control and MANF KO cells were treated with RA for 10, 30, 60, 180, 360, and 720 min. Protein was extracted from cell lysates and subjected to immunoblot. The size of the proteins (kDa) was labeled next to each band. **(B)** MANF protein levels were quantified and normalized with β-actin. **(C)** p-Akt protein levels were quantified and normalized with total Akt. **(D)** p-Erk protein levels were quantified and normalized with total Erk. **(E)** p-mTOR protein levels were quantified and normalized with total mTOR. **(F)** p-P70S6 protein levels were quantified and normalized with total P70S6. The data were expressed as the mean ± SEM of three independent experiments and were analyzed by Two-way ANOVA followed with the Bonferroni’s *post hoc* test, *P < 0.05, **P < 0.01, ***P < 0.001.

**FIGURE 7:**
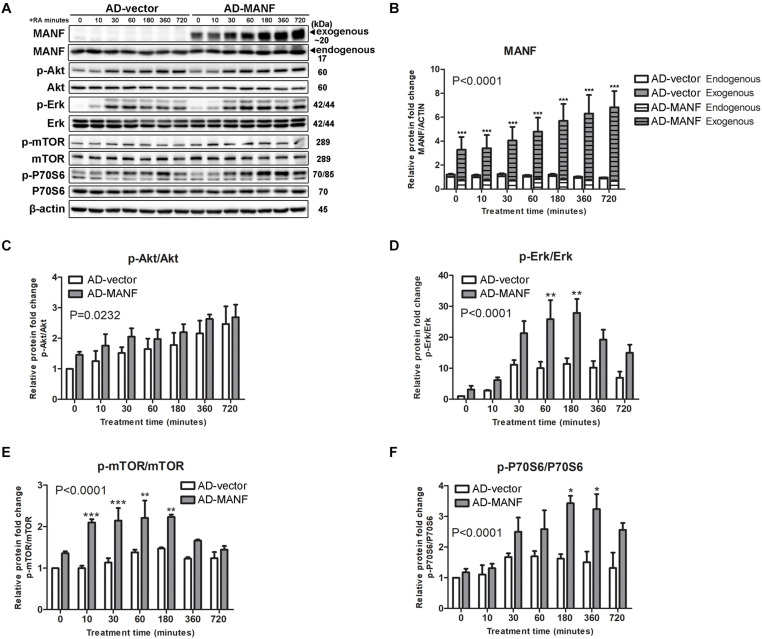
MANF overexpression enhances RA-induced activation of Akt/mTOR and Erk/mTOR signaling pathways. **(A)** AD-vector and AD-MANF transduced cells were treated with RA for 10, 30, 60, 180, 360, and 720 min. Protein was extracted from cell lysates and subjected to immunoblot. The size of the proteins (kDa) was labeled next to each band. **(B)** Endogenous and exogenous MANF protein levels were quantified and normalized with β-actin. **(C)** p-Akt protein levels were quantified and normalized with total Akt. **(D)** p-Erk protein levels were quantified and normalized with total Erk. **(E)** p-mTOR protein levels were quantified and normalized with total mTOR. **(F)** Quantification of p-P70S6 protein levels were quantified and normalized with total P70S6. The data were expressed as the mean ± SEM of three independent experiments and were analyzed by Two-way ANOVA followed with the Bonferroni’s *post hoc* test, *P < 0.05, **P < 0.01, ***P < 0.001.

Since Akt/mTOR and Erk/mTOR are key regulators for neurite outgrowth, we then tested whether these signaling cascades were involved in the role of MANF in regulating neurite outgrowth. We first examined changes in the expression of Akt/mTOR and Erk/mTOR cascades genes in control and MANF KO cells, and AD-vector and AD-MANF transduced cells within 12 h of RA treatment. As expected, in control and AD-vector transduced cells, the initiation of RA-induced neurite outgrowth was correlated with an upregulation of Akt/mTOR and Erk/mTOR signaling pathways. We observed a steady 2-fold increase of p-Akt in control cells by the end of the 12 h RA-treatment (Figures 6A,C). The expression of p-Erk in control cells peaked at 1–3 h to 11-fold and then started to decline (Figures 6A,D). Following the activation of Akt and Erk, the phosphorylation of mTOR and P70S6 in control cells was also increased to 1.4-fold at 3 h and 2-fold at 6 h, respectively, and then started to decline (Figures 6A,E,F). Similar Akt/mTOR and Erk/mTOR activation was observed in RA-treated AD-vector transduced cells (Figure 7). Strikingly, RA-treated MANF KO cells exhibit significantly less extent of Akt and Erk activation compared to control cells. RA failed to induce the phosphorylation of Akt in MANF KO cells (Figures 6A,C) and the induction of p-Erk was significantly reduced from 11-fold in control cells to only 6-fold in KO cells (Figures 6A,D). As a result, the level of p-mTOR and p-P70S6 was also reduced in MANF KO cells (Figures 6A,E,F). On the contrary, AD-MANF transduced cells overexpressing intracellular MANF (Figure 7A) showed significantly elevated expression of p-Akt and p-Erk compared to AD-vector transduced cells (Figures 7A,C,D). p-mTOR and p-P70S6 were also significantly upregulated in MANF overexpressed N2a cells (Figures 7B,E,F).

**FIGURE 8:**
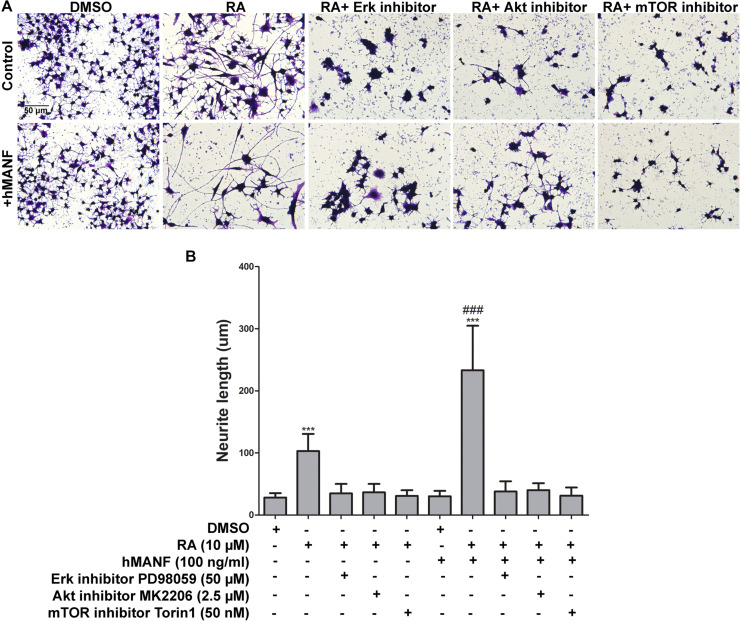
Pharmacological inhibition of Akt, Erk, and mTOR blocks MANF-enhanced neurite outgrowth in response to RA. **(A)** Representative images of N2a cells treated with DMSO or RA or RA+inhibitors for 3 days with or without addition of recombinant hMANF (100 ng/ml). Cells were fixed and stained with crystal violet for visualization. **(B)** Average neurite length was measured, and analyzed by One-way ANOVA followed with the Tukey’s *post hoc* test, ***P < 0.0001 compared to DMSO treated cells, ^###^*P* < 0.0001 compared to RA treated cells. The data were expressed as the mean ± SEM of three independent experiments.

To further confirm that MANF facilitates RA-induced neurite outgrowth via activating Erk/mTOR and Akt/mTOR signaling pathways, control cells were treated with RA and MANF at the presence of Erk inhibitor PD98059 (50 μM), Akt inhibitor MK2206 (2.5 μM), and mTOR inhibitor Torin 1 (50 nM), respectively. PD98059 is a highly selective MEK1 inhibitor, inhibiting the phosphorylation Erk1/2. MK2206 binds to and inhibits the phosphorylation of Thr308 and Ser 473 of Akt, resulting in the inhibition of the PI3K/Akt signaling pathway. Torin 1 is a strong inhibitor for both mTOR-containing complexes, mTORC1 and mTORC2. Treatment of either inhibitors could cause reduced cell proliferation and apoptosis. Dosages for each inhibitor were tested and the minimum dosage that was not toxic to the cells (data not shown) and showed effective inhibition of the phosphorylation of Erk, Akt, and mTOR (Supplementary Figure S3), respectively, was used. As expected, the neurite of RA-stimulated N2a cells reached 103.01 ± 27.63 μm after 3 days of treatment. The addition of Erk, Akt, or mTOR inhibitors significantly reduced RA-induced neurite to 34.74 ± 15.32, 36.39 ± 13.77, and 30.75 ± 9.17 μm, respectively (Figure 8). More importantly, the inhibitors also eliminated the effect of MANF in enhancing RA-induced neurite outgrowth. When treated with RA plus MANF, the average neurite length reached to 233.13 ± 71.71 μm; while the addition of Erk, Akt, or mTOR inhibitors reduced the average neurite length to 37.84 ± 16.46, 40.09 ± 11.16, and 31.27 ± 13.09 μm, respectively, which were significantly shorter compared to RA plus MANF alone (Figure 8).

**FIGURE 9:**
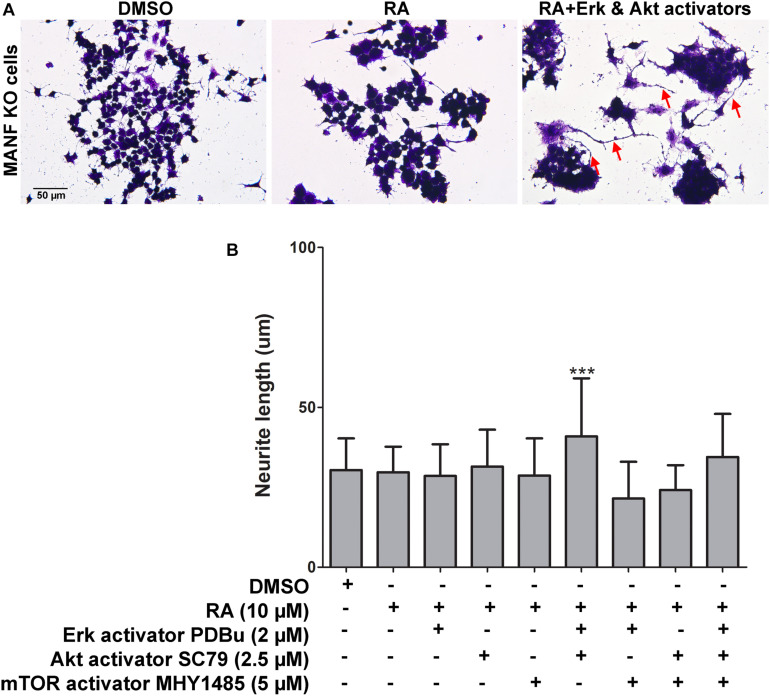
Pharmacological activation of Akt, Erk, and mTOR partially rescues neurite outgrowth defects in MANF KO cells in response to RA. **(A)** Representative images of MANF KO cells treated with DMSO or RA or RA+Akt and Erk activators for 3 days. Cells were fixed and stained with crystal violet for visualization. Red arrows indicate cells with neurite outgrow in response to RA treatment. **(B)** Average neurite length was measured, and analyzed by One-way ANOVA followed with the Tukey’s *post hoc* test, ***P < 0.0001. The data were expressed as the mean ± SEM of three independent experiments.

Next, we tested whether treatment of Erk, Akt, and mTOR activators can rescue the neurite outgrowth defects in MANF KO cells in response to RA treatment. Erk activator PDBu (2 μM), Akt activator SC79 (2.5 μM), and mTOR activator MHY1485 (5 μM) were used at the dosage which was not toxic to the cells and showed effective activation of the phosphorylation of Erk, Akt, and mTOR, respectively (Supplementary Figure S3). PDBu is a potent activator of protein kinase C (PKC), which further activates Erk as a downstream PKC target. SC79 inhibits Akt membrane translocation and enhances Akt phosphorylation. MHY1485 is a cell-permeable activator of mTOR that has been shown to increase the phosphorylation of mTOR at Ser2448. We found that when treated separately, none of the activators was able to rescue the phenotype (Figure 9B). Considering that it might not be sufficient for the neurite to grow by activating just one of the signaling pathways, we then treated the cells with two or all three activators simultaneously. Interestingly, when treated with Erk and Akt activators together, we observed a slight but significant increase in the neurite length in some of the MANF KO cells from 30.38 ± 9.89 to 40.92 ± 18.16 μm (Figures 9A,B). However, none of the other combinations of treatments can rescue the defects of RA-induced neurite outgrowth in MANF KO cells (Figure 9B).

### MANF Deficiency Impairs Protein Synthesis

**FIGURE 10:**
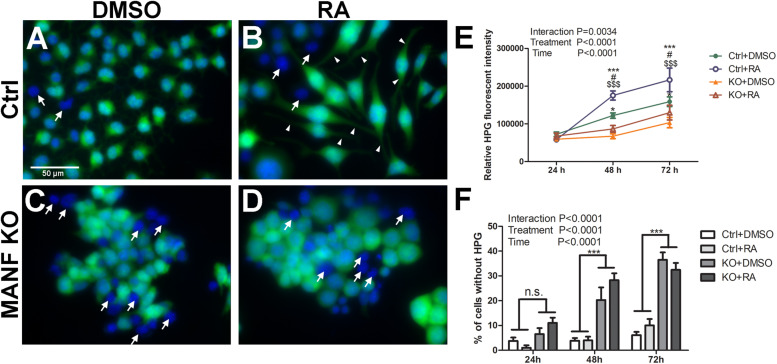
Protein synthesis is reduced in MANF KO cells. Control cells treated with DMSO **(A)** or RA **(B)** and then incubated with HPG. MANF KO cells treated with DMSO **(C)** or RA **(D)** and then incubated with HPG. White arrows indicate cells without HPG signal (cells lacking nascent protein synthesis); white arrow heads indicate neurites with HPG signal. **(E)** HPG fluorescence intensities in control and MANF KO cells were quantified and analyzed by Two-way ANOVA followed with the Bonferroni’s *post hoc* test. ^#^*P* < 0.05 compared to Ctrl DMSO; *P < 0.05, ***P < 0.001 compared to KO DMSO; ^$$$^*P* < 0.001 compared to KO RA. The data were expressed as the mean ± SEM of three independent experiments. **(F)** Percentage of cells without HPG signal was quantified, and analyzed by Two-way ANOVA followed with the Bonferroni’s *post hoc* test, ***P < 0.0001, n.s. not statistically significant. The data were expressed as the mean ± SEM of three independent experiments.

Protein synthesis is critical for neurite formation and can be regulated by the activation of Akt/mTOR and Erk/mTOR signaling pathways. Since our data suggests that MANF positively regulates Akt/mTOR and Erk/mTOR signaling, we sought to further test whether protein synthesis was affected in MANF KO cells and whether it was involved in its defect in RA-induced neurite outgrowth. HPG protein synthesis assay revealed that N2a cells had robust protein synthesis. Fluorescent imaging revealed that nascent protein was mainly localized in the cell soma in DMSO treated control cells (Figure 10A). For RA treated control cells, fluorescent signal was also detected in the neurites, indicating that nascent protein was also present in the growing neurites (Figure 10B, arrowheads). MANF KO cells also exhibit robust fluorescent signal (Figure 10C), but it was limited to the cell soma in both DMSO and RA treated groups, indicating a lack of neurite outgrowth in response to RA (Figure 10D). Quantification of the fluorescent intensities revealed that for both control and KO cells, RA treatment resulted in a higher rate of protein synthesis compared to DMSO treatment, and it increased over time (Figure 10E). However, MANF KO cells have significantly less fluorescent signal compared to control cells. In addition, we noticed that regardless of RA treatment, a small portion (<5% at 24 and 48 h, ∼10% at 72 h) of control cells showed no nascent protein synthesis during the time of the assay (Figures 10A,B, arrows). MANF KO cells, on the other hand, showed significantly higher percentage of cells (∼10% at 24 h, ∼20% at 48 h, ∼35% at 72 h) that were undetectable for nascent protein (Figure 10C,D, arrows). These data suggest that MANF deficiency impairs N2a cell protein synthesis, which may further contribute to the neurite outgrowth defects in MANF KO cells.

## Discussion

Neurite outgrowth is a critical process for neuron differentiation and regeneration in neurological diseases. In this study, we demonstrated that MANF was necessary for RA-induced neurite outgrowth by positively regulating Akt/mTOR and Erk/mTOR signaling pathways. Neurite outgrowth requires protein synthesis, which is regulated by Akt/mTOR and Erk/mTOR signaling pathways (Read and Gorman, 2009; Doherty et al., 2000). We are the first to show that MANF regulates neurite outgrowth through the regulation of protein synthesis and the activation of Akt/mTOR and Erk/mTOR signaling pathways.

Neuro2a and SH-SY5Y cells are common neuronal cell line models widely used for studying neuronal differentiation *in vitro* due to their ability to proliferate and to be easily transfected compared to primary neuron. Neuronal cell lines can be induced into neuron-like cells, where they turn on the expression of neuronal cell markers and grow neuronal processes resembling axons and dendrites. However, as many neuronal cell lines were derived from immortalized neuronal tumors and have undergone numerous proliferations that could accumulate mutations and may not recapitulate the properties of normal neurons from the nervous system. We used neuronal cell lines N2a and SH-SY5Y to show that RA treatment induced neurite outgrowth and MANF deficiency attenuated this process. It is noted that a cell with longer neurites seems also to present enlarged cell bodies with increased neurite caliber. This suggests that RA treatment may also cause general growth and hypertrophy of the cells. It is essential to confirm cell line results with primary neurons. The role of MANF in neurite extension has been investigated in primary neurons and neural precursors as well as in animals. A study by Ko et al. reported that MANF was required for the neurite extension and maintenance in cultured retinal ganglion cells (RGCs) isolated from 3 to 5 days old rat retina (Ko et al., 2020). Another study by Tseng et al., 2017a reported that complete conventional MANF knockout mice exhibit impaired neurite outgrowth *in vivo*, and MANF-deficient neuronal stem cells isolated from the embryonic day 13.5 (E13.5) knockout mice have defect in neurite extension when cultured and differentiated *in vitro* (Tseng et al., 2017a). However, these studies did not examine the underlying cellular/molecular mechanisms. Our study used neuronal cell lines to confirm the findings and further investigate cell signaling pathways responsible for MANF’s effect.

We used both siRNA and CRISPR to generate MANF deficient cells and showed that loss of MANF attenuates the ability of N2a and SH-SY5Y cells to grow neurites in response to RA-stimulation. These methods are complementary. There are two advantages of generating stable MANF knockout using CRISPR: (1) As the effectiveness of siRNA is usually diluted out around 3–4 days after transfection due to cell proliferation, the generation of stable MANF knockout cells allow us to perform experiments with a longer time span, such as the examination of neurite outgrowth with 6 days of RA treatment, which provided evidence of MANF knockout indeed attenuates but not just delays RA-induced neurite outgrowth; (2) The efficiency of siRNA transfection varies depending on the condition of cells and transfection reagents. For example, immunoblotting analysis confirmed that siRNA method only reduced MANF by 50%. MANF knockout using CRISPR had a much better efficiency. However, stable MANF knockout using CRIPSR on the other hand, may trigger compensatory expression of other UPR genes, and transient knock out of MANF by siRNA is able to overcome this shortcoming.

Different from most neurotrophic factors that are secreted, MANF is largely retained in the lumen of the ER and is secreted under pathological conditions where ER stress is elevated (Apostolou et al., 2008; Tadimalla et al., 2008; Glembotski et al., 2012; Oh-Hashi et al., 2012; Henderson et al., 2013). Under normal condition, MANF is retained in the ER via its calcium-dependent interaction with GRP78 (glucose-regulated protein 78 kDa, BiP) in the lumen of the ER and the binding with endoplasmic reticulum protein retention (KDEL) receptors located on Golgi apparatus and cell surface (Glembotski et al., 2012; Henderson et al., 2013). In pathological conditions with elevated ER-stress, reduced ER calcium level interferes with the binding of MANF and GRP78. ER-stress also induces protein transportation from the ER to Golgi, resulting in an accumulation of MANF and GRP78 in the lumen of Golgi. GRP78 competes with MANF on the binding with KDEL receptors, leading to a decreased retention of MANF in the ER. MANF has been implicated as a neurotrophic factor that regulates neuron development and protects neuron from various pathological conditions (Li-Na et al., 2017; Lindahl et al., 2017). The mechanisms involved in the neuroprotective role of MANF remains unclear, although increasing evidence has suggested the involvement of MANF in ameliorating ER-stress induced apoptosis (Lindahl et al., 2017; Xu M. et al., 2018; Zhang et al., 2017a; Zhang et al., 2017b; Zhu et al., 2016). There are inconsistent reports regarding the role of intracellular and extracellular MANF in the context of neuroprotection. For example, extracellular application of MANF was reported to protect neurons and myocardial cells from ischemic injuries (Tadimalla et al., 2008; Airavaara et al., 2009; Glembotski et al., 2012), while MANF had to be injected directly into neurons to protect against Bax-dependent apoptosis or overexpressed intracellularly in Hela cells to improve cell viability under glucose-free conditions and tunicamycin treatment (Apostolou et al., 2008; Hellman et al., 2011). We showed that either overexpression of MANF intracellularly by adenovirus or addition of recombinant hMANF into cell culture can induce longer neurite outgrowth in response to RA stimulation. However, the addition of MANF in the culture media did not rescue neurite growth in MANF KO cells, indicating that the intracellular MANF plays an important role that is not compensated by MANF in the culture media. One of the limitations of this study is that we were not able to exclude the secretion of adenovirus-mediated MANF from cells, which makes it difficult to distinguish the function of intracellular and extracellular MANF. To further study the function of MANF in these two forms, we need to determine if the intracellularly overexpressed MANF can also increase the level of MANF being secreted and if blocking MANF secretion will affect the role of MANF in facilitating RA-induced neurite outgrowth.

PI3K/Akt and Ras/Erk pathways are important intracellular signal transduction cascades that are critical for fundamental cellular functions, regulating cell proliferation, growth, survival, mobility and cell death (McCubrey et al., 2007; Mendoza et al., 2011). They are often dysregulated in human cancers, leading to aberrant activation of the signaling cascades (Asati et al., 2016). Disturbed activation of the PI3K/Akt/and Ras/Erk pathways are oncogenic, enhancing the growth, survival, and metabolism of cancer cells (Jokinen and Koivunen, 2015). PI3K/Akt and Ras/Erk signaling pathways are also key mediators for neuronal survival and several aspects of neurite outgrowth, including cell skeleton reorganization and stabilization, neurite branching and extension, and axon formation (Frebel and Wiese, 2006; Read and Gorman, 2009; Hausott and Klimaschewski, 2019). Mammalian target of rapamycin (mTOR) is one of the major downstream effectors of PI3K/Akt and Ras/Erk signaling pathways (Potter et al., 2002; Ma et al., 2005). mTOR is a serine/threonine protein kinase which is a key regulator for cell growth and metabolism by controlling protein translation and lipogenesis (Takei and Nawa, 2014). It promotes protein synthesis through the phosphorylation of P70S6 kinase, which in turn phosphoactivates the ribosomal protein S6 and lead to increased mRNA and protein synthesis (Laplante and Sabatini, 2009). Protein synthesis is essentially required for neurite outgrowth. Increased requirement of new protein synthesis is accompanied with the process of neurite initiation, branching, elongation, and stabilization (Tojima and Ito, 2004; Flynn, 2013). Protein synthesis is important for filopodia and neurite formation (Gallo, 2013; Sainath and Gallo, 2015). Local protein synthesis is critical for axon elongation and pathfinding (Twiss and van Minnen, 2006). Decreased protein synthesis has been reported in various neurodegenerative disorders, such as Alzheimer’s disease (Chang et al., 2006). The activation of PI3K/Akt/mTOR and Ras/Erk/mTOR signaling cascades positively regulates protein synthesis, which is critical for cell differentiation and neurite outgrowth in neurons (Fujii et al., 1982; Takei and Nawa, 2014; Schanzenbacher et al., 2016; Rozenbaum et al., 2018).

Several studies have suggested that MANF can activate Akt and Erk signaling pathways. In an *in vitro* study, extracellular MANF was shown to protect human neuroblastoma SH-SY5Y cells from 6-hydroxydopamine (6-OHDA) induced cell death via the activation of PI3K/Akt/mTOR pathway (Hao et al., 2017). In addition to mTOR, several other downstream effectors of the PI3K/Akt signaling pathway including GSK3β, MDM2, and NF-κB have been reported to be activated by treatment of recombinant hMANF in the rodent brain, which was associated with enhanced neuron survival in neurodegenerative diseases and intracerebral hemorrhage models (Hao et al., 2017; Zhang et al., 2017b; Li et al., 2018; Xu M. et al., 2018). MANF has also been reported to activate Erk in a study showing that intracellular MANF overexpression facilitated neuron migration and activated STAT3 and Erk in mice SVZ explant (Tseng et al., 2017a). However, in the same study when NSC cultures were treated with recombinant hMANF, neither Akt nor Erk was activated, suggesting the activation of Akt and Erk by MANF may be cell type- and context-dependent.

We showed that MANF knockout in N2a cells attenuated RA-induced activation of Akt/mTOR and Erk/mTOR, leading to limited neurite outgrowth. MANF overexpression on the other hand lead to longer neurite length which was correlated with increased activation of Akt/mTOR and Erk/mTOR in response to RA. Treatment of Akt, Erk or mTOR inhibitors blocked RA-induced N2a cells neurite outgrowth. Moreover, MANF enhanced neurite outgrowth and Akt/mTOR and Erk/mTOR activation can also be blocked by inhibition of Akt, Erk or mTOR. We observed minimal toxic effect of these inhibitors at the concentrations we used, although inhibitors appeared to slightly reduce cell density. In general, it is easier for N2a cells to extend longer neurite at lower density, however, we observed reduced neurite outgrowth in RA and RA+MANF groups after inhibitor treatments. As a result, we believe that the effect of these inhibitors on neurite outgrowth did not result from the potential toxicity and alterations in cell density, rather than through the activation of Akt/mTOR and Erk/mTOR signaling cascades.

In line with the insufficient activation of Akt/mTOR and Erk/mTOR signaling pathways, we also observed impaired protein synthesis in MANF deficient cells. Consistent with previous report that decreased protein synthesis was also observed in cultured MANF ^–/–^ mice NSCs (Tseng et al., 2017b), our results suggest that the impaired protein synthesis may contribute to the defect of neurite outgrowth in MANF knockout cells.

Our data indicate that MANF facilitates RA-induced neurite outgrowth by positively regulating Akt/mTOR and Erk/mTOR signaling pathways. However, the mechanisms by which MANF activates the Akt/mTOR and ERK/mTOR signaling pathways are currently unknown. In addition, in the experiment of treating MANF KO cells with RA and Akt, Erk or mTOR activators, except for a mild neurite outgrowth in Akt plus Erk activators treated group, none of the other groups showed a rescue in neurite outgrowth. This data indicates that besides Akt/mTOR and Erk/mTOR signaling pathways, there must be other signaling pathways or cellular processes that are affected by MANF knockout and are also important for RA-induced neurite outgrowth. ER stress can be one of the candidate mechanisms, as MANF is known to alleviate ER stress and elevated ER stress is associated with many neurodegenerative diseases and defects in neurite outgrowth (Kawada et al., 2018).

## Conclusion

In conclusion, our study demonstrated that MANF deficiency attenuates RA-induced neurite outgrowth and MANF overexpression facilitates the growth of longer neurites. This is the first study to demonstrate that MANF regulate neurite outgrowth through activating Akt/mTOR and Erk/mTOR signaling pathways and protein synthesis. This study provides evidence that MANF is involved in neuronal differentiation and it may be a potential candidate to facilitate the regeneration of neuronal processes in neurodegenerative diseases.

## Data Availability Statement

All datasets presented in this study are included in the article/Supplementary Material.

## Author Contributions

WW and JL designed the study and contributed to drafting the manuscript. WW, YW, HL, HX, MX, JF, and MM performed the experiment, collated data, and carried out data analyses. All authors have read and approved the final submitted manuscript.

## Conflict of Interest

The authors declare that the research was conducted in the absence of any commercial or financial relationships that could be construed as a potential conflict of interest.

## Supplementary Material

Supplementary Figure S1 Expression of exogenous MANF in N2a cells after adenovirus transduction. **(A)** Protein was extracted from cells transduced with AD-vector and AD-MANF at 24, 48, 72, 96, and 120 h after transduction, then subjected to immunoblot. The expression of HA tag and MANF was examined. β-actin was used as a loading control. The size of the proteins (kDa) was labeled next to each band. **(B)** Exogenous MANF protein levels were quantified as relative levels normalized to β-actin. Two-way ANOVA followed with the Bonferroni’s *post hoc* test, ^∗∗^*P* < 0.01, ^∗∗∗^*P* < 0.0001. The data were expressed as the mean ± SEM of three independent experiments.

Supplementary Figure S2 MANF cannot be reintroduced into MANF KO cells. **(A)** Immunofluorescent images showing control and MANF KO cells were transduced with AD-vector and AD-MANF and then immunolabeled with HA tag (green) and MANF (red). Cell nuclei were stained by DAPI. **(B)** The percentage of cells with HA tag expression was quantified. Student’s *t*-test, ^∗∗∗^*P* < 0.0001. The data were expressed as the mean ± SEM of three independent experiments. **(C)** Protein was extracted from cells 36 h after being incubated with AD-vector and AD-MANF for 1–4 h, and then subjected to immunoblot with HA tag and MANF antibodies. β-actin was used as a loading control. **(D)** Protein was extracted from control and MANF KO cells, and then immunoblot with Cas9 antibody. β-actin was used as a loading control. The size of the proteins (kDa) was labeled next to each band. The experiment was replicated three times.

Supplementary Figure S2 The effect of pharmacological inhibition or activation of Akt, Erk and mTOR on the expression of p-Akt, p-Erk, and p-mTOR in response to RA treatment. **(A)** Quantification of p-Akt, p-Erk, p-mTOR, and p-P70S6 protein expression in control cells treated with DMSO, RA or RA+inhibitors. β-actin was used as a loading control. One-way ANOVA followed with the Tukey’s *post hoc* test, ^∗^*P* < 0.05, ^∗∗^*P* < 0.01 compared to DMSO treated group; ^#^*P* < 0.05, ^##^*P* < 0.01, ^###^*P* < 0.001 compared to RA treated group. The data were expressed as the mean ± SEM of three independent experiments. **(B)** Quantification of p-Akt, p-Erk, p-mTOR, and p-P70S6 protein expression in MANF KO cells treated with DMSO, RA or RA+activators. β-actin was used as a loading control. One-way ANOVA followed with the Tukey’s *post hoc* test, n.s. not statistically significant, ^∗^*P* < 0.05 compared to DMSO treated group; ^#^*P* < 0.05 compared to RA treated group. The data were expressed as the mean ± SEM of three independent experiments.
