# Supplementary figures and images for: Mesencephalic Astrocyte-Derived Neurotrophic Factor (MANF) Regulates Neurite Outgrowth Through the Activation of Akt/mTOR and Erk/mTOR Signaling Pathways
Source: Front Mol Neurosci. 2020 Sep 24;13:560020. doi: 10.3389/fnmol.2020.560020 (PMC7541815; doi:10.3389/fnmol.2020.560020)

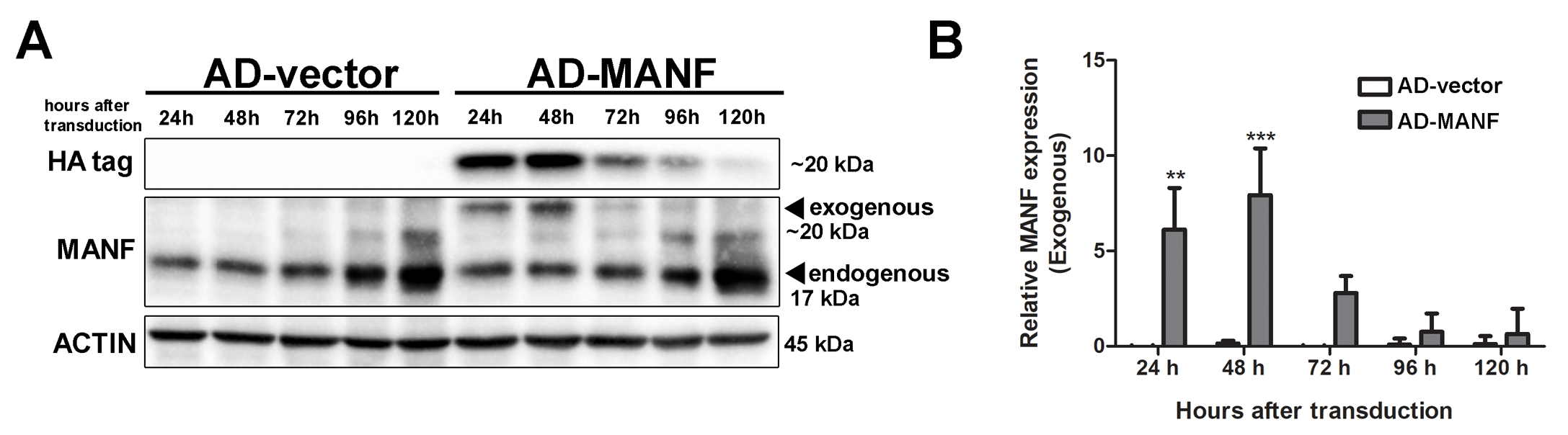

Supplement: Supplementary Figure S1 — Expression of exogenous MANF in N2a cells after adenovirus transduction. (A) Protein was extracted from cells transduced with AD-vector and AD-MANF at 24, 48, 72, 96, and 120 h after transduction, then subjected to immunoblot. The expression of HA tag and MANF was examined. β-actin was used as a loading control. The size of the proteins (kDa) was labeled next to each band. (B) Exogenous MANF protein levels were quantified as relative levels normalized to β-actin. Two-way ANOVA followed with the Bonferroni’s post hoc test, ∗∗P < 0.01, ∗∗∗P < 0.0001. The data were expressed as the mean ± SEM of three independent experiments. [file Image_1.TIF]

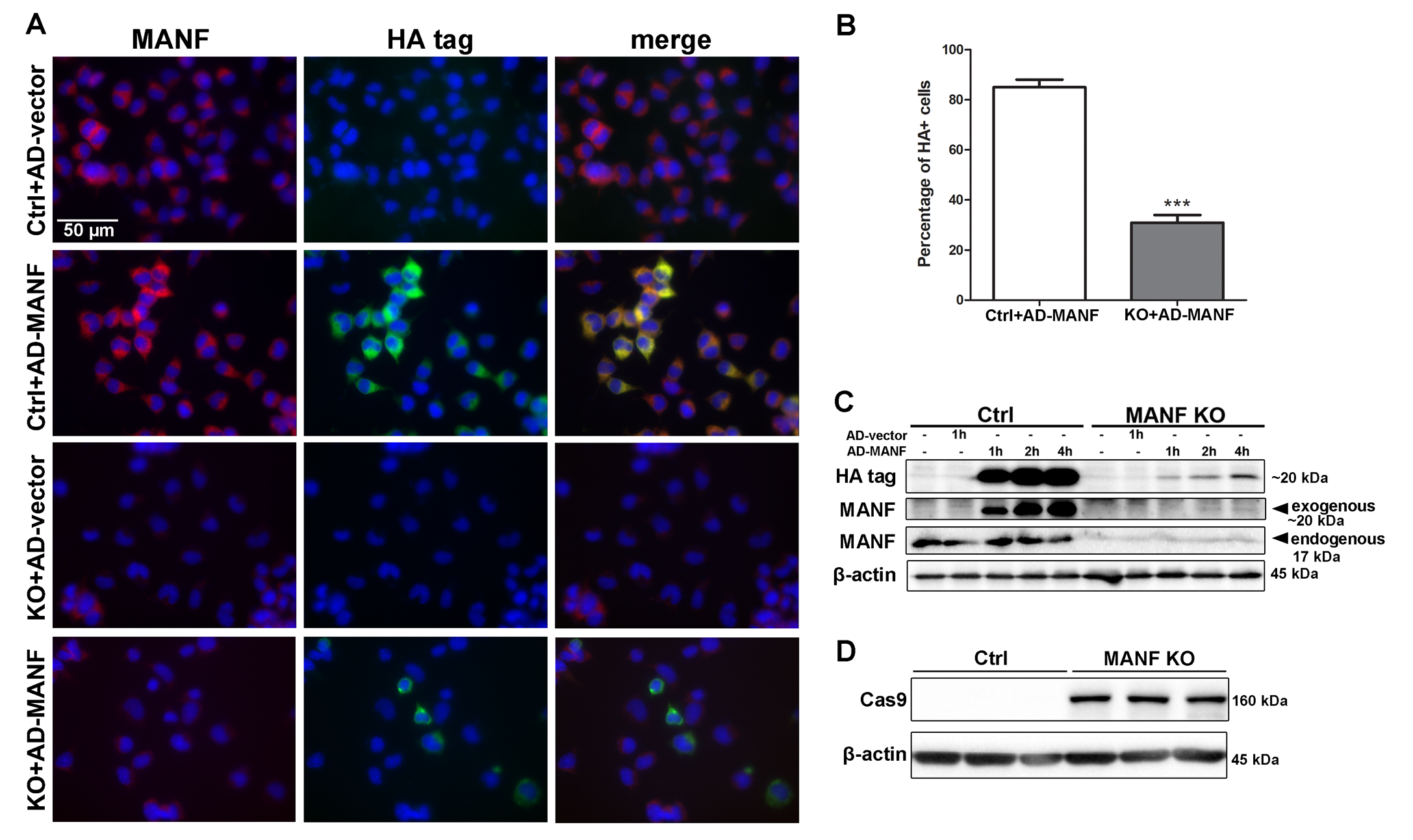

Supplement: Supplementary Figure S2 — MANF cannot be reintroduced into MANF KO cells. (A) Immunofluorescent images showing control and MANF KO cells were transduced with AD-vector and AD-MANF and then immunolabeled with HA tag (green) and MANF (red). Cell nuclei were stained by DAPI. (B) The percentage of cells with HA tag expression was quantified. Student’s t-test, ∗∗∗P < 0.0001. The data were expressed as the mean ± SEM of three independent experiments. (C) Protein was extracted from cells 36 h after being incubated with AD-vector and AD-MANF for 1–4 h, and then subjected to immunoblot with HA tag and MANF antibodies. β-actin was used as a loading control. (D) Protein was extracted from control and MANF KO cells, and then immunoblot with Cas9 antibody. β-actin was used as a loading control. The size of the proteins (kDa) was labeled next to each band. The experiment was replicated three times. [file Image_2.TIF]

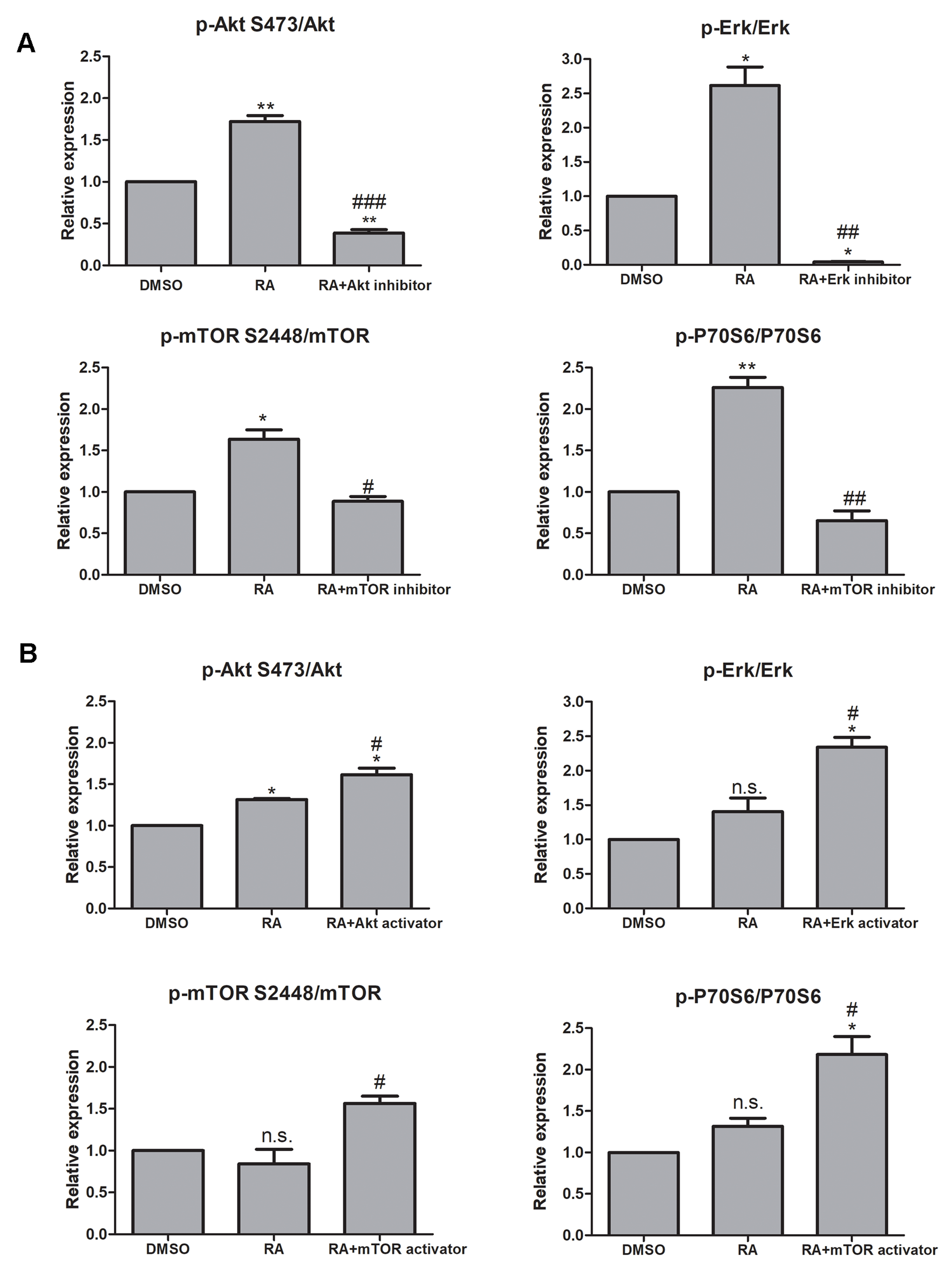

Supplement: Supplementary Figure S2 — The effect of pharmacological inhibition or activation of Akt, Erk and mTOR on the expression of p-Akt, p-Erk, and p-mTOR in response to RA treatment. (A) Quantification of p-Akt, p-Erk, p-mTOR, and p-P70S6 protein expression in control cells treated with DMSO, RA or RA+inhibitors. β-actin was used as a loading control. One-way ANOVA followed with the Tukey’s post hoc test, ∗P < 0.05, ∗∗P < 0.01 compared to DMSO treated group; #P < 0.05, ##P < 0.01, ###P < 0.001 compared to RA treated group. The data were expressed as the mean ± SEM of three independent experiments. (B) Quantification of p-Akt, p-Erk, p-mTOR, and p-P70S6 protein expression in MANF KO cells treated with DMSO, RA or RA+activators. β-actin was used as a loading control. One-way ANOVA followed with the Tukey’s post hoc test, n.s. not statistically significant, ∗P < 0.05 compared to DMSO treated group; #P < 0.05 compared to RA treated group. The data were expressed as the mean ± SEM of three independent experiments. [file Image_3.TIF]
